# Modeling Bainitic Transformations during Press Hardening

**DOI:** 10.3390/ma14030654

**Published:** 2021-01-31

**Authors:** Mingxuan Lin, Carina Zimmermann, Kai Wang, Martin Hunkel, Ulrich Prahl, Robert Spatschek

**Affiliations:** 1 Instituts für Eisenhüttenkunde, RWTH Aachen University, 52072 Aachen, Germany; Ulrich.Prahl@imf.tu-freiberg.de; 2 Leibniz-Institut für Werkstofforientierte Technologien—IWT, 28359 Bremen, Germany; czimmermann@iwt-bremen.de (C.Z.); hunkel@iwt-bremen.de (M.H.); 3 Institut für Energie- und Klimaforschung IEK-2, Forschungszentrum Jülich GmbH, 52425 Jülich, Germany; k.wang@fz-juelich.de (K.W.); r.spatschek@fz-juelich.de (R.S.); 4 Institut für Metallformung, Technische Universität Bergakademie Freiberg, 09599 Freiberg, Germany; 5 JARA-ENERGY, 52425 Jülich, Germany

**Keywords:** bainite, phase field, press hardening

## Abstract

We revisit recent findings on experimental and modeling investigations of bainitic transformations under the influence of external stresses and pre-strain during the press hardening process. Experimentally, the transformation kinetics in 22MnB5 under various tensile stresses are studied both on the macroscopic and microstructural level. In the bainitic microstructure, the variant selection effect is analyzed with an optimized prior-austenite grain reconstruction technique. The resulting observations are expressed phenomenologically using a autocatalytic transformation model, which serves for further scale bridging descriptions of the underlying thermo-chemo-mechanical coupling processes during the bainitic transformation. Using analyses of orientation relationships, thermodynamically consistent and nondiagonal phase field models are developed, which are supported by ab initio generated mechanical parameters. Applications are related to the microstructure evolution on the sheaf, subunit, precipitate and grain boundary level.

## 1. Introduction

The development of an advanced press hardening technology is of high interest in automobile industry because it is an efficient way to produce medium-to-high strength steel parts that can meet the increasing demand of lightweight chassis. During press hardening an austenitized steel sheet is stamped with tools of controlled temperature, inside which austenite transforms to martensite, bainite or ferrite in the rapid cooling cycle under mechanical loads. The fractions of these microconstituents depend on the process parameters and can strongly affect the final properties [1]. As a result, the local microstructure and mechanical properties of the steel parts can be designed through in-tool heat flow management aided by multiphysics numerical simulations. Although existing industrial press hardening processes produce completely martensitic parts, we focus on a bainite-dominated microstructure because it provides a better strength vs. toughness balance, lower risk of delayed fracture, and significantly higher resistance to edge cracking [2], compared to martensitic microstructure. Therefore, it is important to understand bainitic transformation in complex cooling and loading conditions.

The influence of externally applied stress on bainitic transformation was firstly suggested by Bain [3] and investigated in a large variety of steels throughout the past decades [4,5,6]. The bainite formation accelerates significantly in the presence of pre-strain or stresses [1,7,8]. Jepson et al. found that large compressive stresses change the orientation of bainitic “needles” and prompt the alignment of needles preferentially at 45 ${}^{\circ }$ to the stress axis in various medium-to-high carbon steels [5]. The development of electron backscatter diffraction (EBSD) allows detailed investigation into these variant selection effects [9,10]. Tensile stresses also increase both the length and width of the bainite sub-units significantly [11,12,13]. On the other hand, deformation of the austenite prior to bainitic transformation leads to much finer bainite [14], shorter incubation time [1] and higher strength [15]. Moreover, there occurs a transformation-induced anisotropic eigenstrain at the same direction of the applied stress [8,11]. The transformation plastic strain is a result of variant selection by the externally applied stress, which is known as Magee effect for martensitic transformation [16,17,18]. Theoretical interpretations of these effects are normally constructed analogously to the models of transformation induced plasticity (TRIP) and shape memory effect of martensitic transformation [17]. However, there exist very few quantitative models for simulation of the kinetics and stress-strain response of bainitic transformation under complex thermo-mechanical conditions. Furthermore, techniques of prior-austenite grain reconstruction from EBSD data developed in the past decade [19,20,21] dramatically facilitates experimental investigation of the variant selection effect. In this article we connect experimental observations of the reconstructed austenite grains to the chemo-mechanical models on the mesoscopic length scale.

There is a long controversy whether the bainitic transformation kinetics should be described by a diffusion-controlled model or a diffusionless (autocatalytic-nucleation controlled) model, see [22] and references therein. According to the controversy different models exist, which describe the (stress and pre-strain free) austenite to bainite transformation [14,23,24,25,26]. Various kinetic models exist [9,27,28,29], which use mean-field descriptions of free energies of austenite and bainitic ferrite, with or without the contribution of mechanical energy, which is formulated as the product between the stress and the “shape strain”. These kinetic models only consider the one-way effect of stress on the phase transition because the kinetic equations only describe the evolution of individual bainitic variants. Mahnken et al. [18,30] developed a fully-coupled multi-scale model, including plasticity, heat conduction and bainitic transformation at different length scales. At their lowest scale, 24 scalars are used to represent the volume fractions of all possible bainitic variants inside an austenite grain. Among the few full-field models of bainitic transformation, Arif et al. [31] employed a shape-dependent energy in a phase field model to simulate the mechanical effect on the growth of bainitic sub-units. Song et al. approximated the needle morphology of bainitic ferrite sheaves with anisotropic interface parameters [32,33]. At a similar scale, we also demonstrated earlier a phase field model coupled with linear elasticity [34]. In recent years, the fast development of phase field approaches for dislocation slip [35], martensitic transformation [36,37] and their interactions [38] opens up the opportunity to model bainitic transformation in wide range of scales from various aspects, linking different chemical and mechanical processes. However, despite this progress there is still a strong demand for descriptions of the bainite transformation on all scales to fully capture all relevant processes as a requirement for the development of a digital twin of experimental investigations of the bainitic press hardening process.

In this article we summarize our recent results and methodological progress relevant to the stress-affected bainitic transformation in order to draw a road map for chemo-mechanical modelling of the bainitic press hardening process. The quantitative description of microstructure evolution in this process requires the development of novel state of the art modeling techniques to capture correctly the interaction of phase transformation kinetics and thermodynamics, elastic and plastic deformations as well as chemical effects due to alloying. They provide a basis for the understanding of the phase transformation kinetics under full consideration of the thermo-chemo-mechanical coupling on all length scales. Selected sub-models are benchmarked against alternative modeling descriptions. A subset of the software tools for calculation and visualisation of different aspects of bainitic transformation are summarized in a bainite toolbox available to the readers upon request. In particular, we demonstrate: (1) On the smallest scale ab initio methods can be used for a parameter free prediction of elastic parameters with an extension towards large deformations. A scale bridging inspection with a comparison to classical density functional theory and phase field crystal methods allows to obtain a thorough understanding and quantitative analytical description of stress-strain relations, which can serve as a basis in higher scale modeling of microstructure evolution modeling. (2) The in-depth analyses of EBSD-measured microstructural data concerning the crystallography of the bainitic transformation allows to extract mathematical descriptions of transformation plasticity as a result of orientational-variant selection by applied stresses. Moreover, we extract the variant selection mechanisms which are central for the formulation of mesoscopic descriptions. (3) The proximity to interfaces, e.g., ferrite-austenite interfaces or grain boundaries, can affect the local thermodynamics of carbide precipitation via an elastic interaction. We demonstrate how these scale bridging effects can be formulated effectively for the use of the equilibrium properties and precipitation kinetics of carbides near surfaces and interfaces under the influence of external and internal stresses. (4) Thermodynamically consistent phase field descriptions using non-diagonal interactions allow to link the descriptions quantitatively and consistently to sharp interface models also in the range of finite diffusional contrasts in the phases.

Based on the above phase field model developments we investigate the role of mechanical stresses on the phase transformation kinetics on the carbide precipitate, subunit and sheaf packet level, with a controlled consideration of elastic and plastic interactions, which serves as an interface to macroscopic descriptions of the anisotropic transformation plasticity which is extracted from experimental investigations.

We start with an overview on experimental investigations in Section 2.1, with a focus on the influence of stresses and pre-strain on the bainite transformation kinetics for the steel grade 22MnB5 and investigations of macroscopic properties and microstructural changes. The experimental results are used as a basis for scale bridging modeling descriptions of the underlying strong thermo-chemo-mechanical coupling in Section 2.2. On the largest scale, macroscopic models as phenomenological descriptions of the experimental data are summarized, serving as a further basis for microstructural models. Phase field approaches are widely used here for hierarchical model developments and descriptions ranging from the evolution of bainitic sheaves, subunits and precipitates, down to grain boundary effects. Central results of our work and the present paper are discussed in Section 3. Besides the application of the results in the following section with a focus to the application to the bainitic transformations under the influence of external stress, we emphasize that the developed approaches and tools are also of use for other metallurgical processes.

## 2. Results

### 2.1. Experiments

In this section we briefly review central experiments related to bainitic transformations under applied stresses, which are essential for the understanding of the bainitic press hardening process and the development of multiscale descriptions of the microstructural evolution, focusing on the steel 22MnB5. In addition, the evaluation of experimental EBSD measurements with respect to austenite reconstruction and analysis is shown.

#### 2.1.1. Experimental Onset

Experiments on stress-dependent bainite formation were carried out on specimens taken from cold-rolled sheets with a thickness of 1.8 mm of 22MnB5. The average chemical composition was determined by optical emission spectroscopy and is 0.25% C, 0.22% Si, 1.22% Mn, 0.04% Al, 0.03% Ti, 0.12% Cr and 0.003% B. Flat tensile specimens with a total length of 120 mm (gauge length 48 mm) were taken by wire erosion. The tests were performed using a thermomechanical simulator Gleeble 3500 with an enhanced gas cooling device. Both, length and width of the specimens were measured by laser extensometers during the tests. The specimens were heated up within 1.5 min to austenitizing temperature of 950 ${}^{\circ }C$ and were held there for 5 min. Afterwards the specimens were gas quenched to isothermal transformation temperatures of 450 ${}^{\circ }C$, 500 ${}^{\circ }C$, or 550 ${}^{\circ }C$. At the end of the quenching, stress from 0 to 250 MPa was applied. After the full bainite transformation, the specimens were cooled to ambient temperature. The specimens were analyzed extensively by standard metallography and by EBSD. For the EBSD-measurements a scanning electron microscope (TESCAN VEGA II) equipped with EDX and an EDAX TEAM Pegasus analysis system was used.

#### 2.1.2. Experimental Results

**Figure 1:**
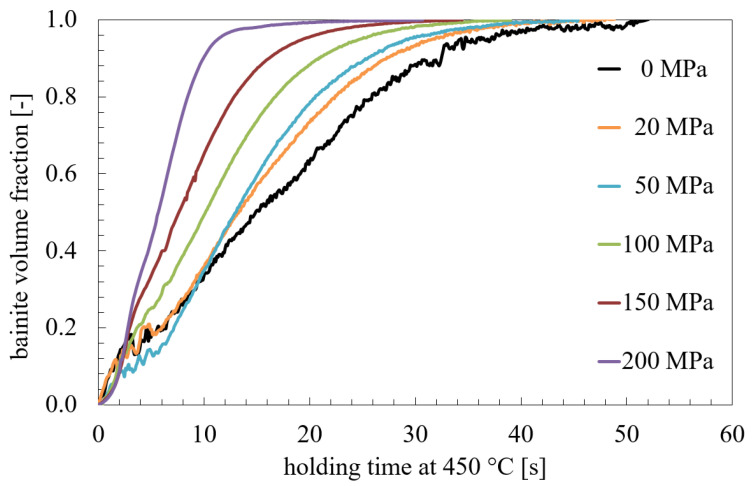
Kinetics of the bainitic transformation in regard of applying different stresses [39].

**Figure 2:**
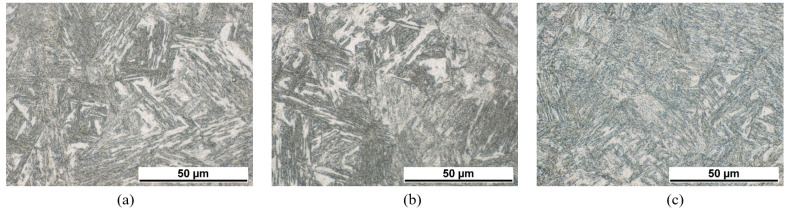
Metallographic micrographs of bainite structure of 22MnB5 after transformation at 450 ${}^{\circ }C$ under applied stresses — (**a**) 0 MPa, (**b**) 50 MPa, (**c**) 200 MPa.

**Figure 3:**
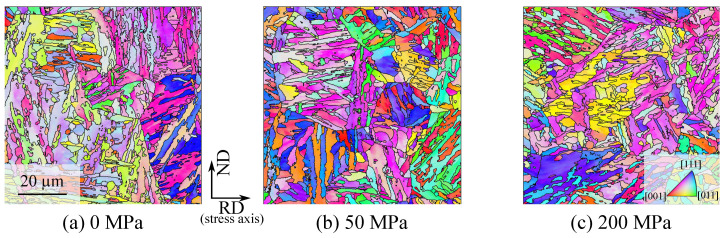
EBSD results of the bainitic transformations at 450 ${}^{\circ }C$ under applied stresses — (**a**) 0 MPa, (**b**) 50 MPa, (**c**) 200 MPa.

The influence of stresses and pre-strain on the bainite transformation kinetics for 22MnB5 are discussed in detail in [39]. The experiments show a strong nonlinearity of the transformation plasticity of the bainite transformation and differences between tension and pressure cases. The effect on the bainitic transformation of various degrees of applied stresses in the range of 0 to 250 MPa, various degrees of deformation in the range of 0 to 18% and the transformation plasticity effect are investigated in the work in [40]. For example, in Figure 1 the influence of applied stress on the bainite transformation kinetics is shown [39]. The applied stress accelerates the transformation significantly. Metallographic determined bainite structures of 22MnB5 transformed at 450 ${}^{\circ }C$ under applied stresses of 0 MPa, 50 MPa, and 200 MPa are shown in Figure 2 and corresponding EBSD-results are shown in Figure 3. Yield strength of austenite is approximately 100 MPa at 450 ${}^{\circ }C$ [39]. Therefrom, results shown in Figure 2a,b and Figure 3a,b are transformed with an applied stress below the yield strength and results shown in Figure 2c and Figure 3c are above the yield strength, respectively. The bainite sheaves have a length of several 10 μm. The more stress is applied in the system the finer the bainite structure becomes.

#### 2.1.3. EBSD Analysis

**Figure 4:**
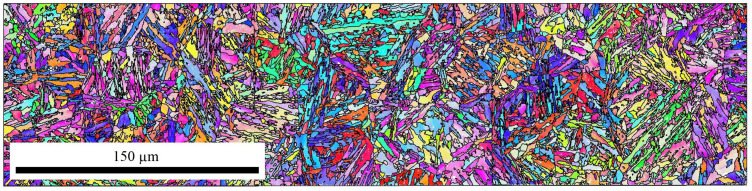
EBSD measurements — 0 MPa.

**Figure 5:**
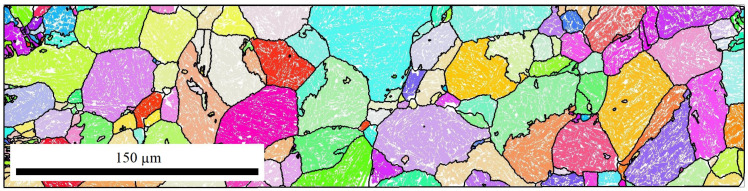
Reconstructed austenite grains — 0 MPa.

**Figure 6:**
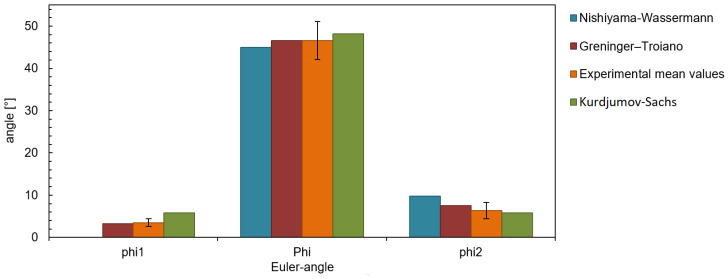
Bainite misorientation corresponding to individual (reconstructed) austenite grains locations, mean values against theoretical orientation relationships — 0 MPa.

**Figure 7:**
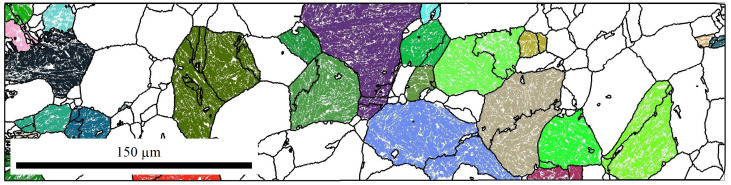
Twins of the reconstructed austenite grains — 0 MPa.

The raw data of the EBSD measurements were further analyzed with the help of the MTEX toolbox for Matlab [41]. Figure 4 shows exemplary the bainite sheaves of a sample without an applied stress (0 MPa). Approximately 10,000 bainitic ferrite grains were detected. From the determined EBSD-data of bainite, the prior-austenite grains (PAGs) were reconstructed using the algorithm by Nyssönen [42,43] and the Kurdjumov-Sachs orientation relationship. The reconstructed PAGs are shown in Figure 5, and the mean PAG size is about 50 μm. Knowing the bainite grains belonging to a single PAG, the real orientation relationship and the austenite orientation were determined by a least square fit. An independent fitting was done for each austenite grain of a size larger than 10 μm. In total, 48 austenite grains were used for the analysis of the orientation relationship. Different orientation relationships are defined as follows [44]: Greninger-Trojano (G-T): ${\left[110\right]}_{\gamma }$ is $2.5{}^{\circ }$ from ${\left[111\right]}_{\alpha }$; Kurdjumov-Sachs (K-S): ${\left[110\right]}_{\gamma }{\|\left[111\right]}_{\alpha }$; Nishiyama-Wassermann (N-W): ${\left[211\right]}_{\gamma }{\|\left[011\right]}_{\alpha }$ and for all ${\left(111\right)}_{\gamma }{\|\left(011\right)}_{\alpha }$ applies. In Figure 6, it is clearly shown that the Greninger-Trojano orientation relationship fits better with the mean values of the experimental data than the Nishiyama-Wassermann and Kurdjumov-Sachs orientation relationships do, similarly to previous studies [45,46,47]. Furthermore, a twinning analysis was applied on the reconstructed PAGs. Two neighbouring austenite grains are considered as twins if their misorientation (rotation) is 60${}^{\circ }$${\langle 111\rangle }_{\gamma }$ with a discrepancy below 2${}^{\circ }$. In Figure 7, all twin-related neighbouring PAGs were displayed in the same colour, which indicates a significant amount of austenite twins and will be discussed in Section 2.2.2 and Section 3.

#### 2.1.4. Multifaceted Correlative Microstructure Characterization

**Figure 8:**
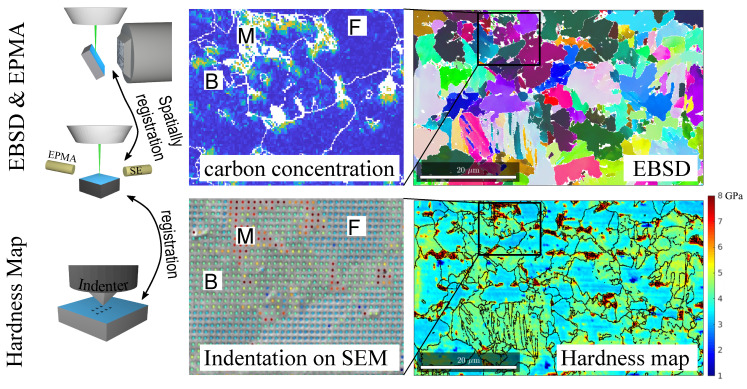
Multifaceted correlative characterization of a complex-phase microstructure (CP800) comprising bainite (B), ferrite (F) and small islands of martensite (M). The hardness map of the microstructure is constructed from around 20,000 nano-indentation points. After spatial registration between various maps, the grain boundaries are overlaid on the hardness map. The carbon concentration map is measured with electron probe microanalyzer (EPMA) where the color code represents the corresponding EPMA signal intensity.

The press hardened microstructure may comprise multiple microconstituents, including bainite as the matrix, martensite and polygonal ferrite. Their volume fraction and morphology are the primary factors that influence the mechanical properties. A mixture of these 3 microconstituents of vastly different hardness has been successfully applied to a family of commercial steel grades known as complex phase steels. The relation between local microstructural features and local mechanical properties was explored with correlative characterization, a systematic method to correlatively analyze the multifaceted microstructural datasets collected in SEM (with EBSD and electron probe microanalyzer) and nano-hardness scanner [48,49]. Figure 8 demonstrates the correlative characterization approach with results of a commercial complex phase steel grade, CP800.

### 2.2. Modeling and Simulation

In this section we summarize modeling descriptions of bainitic microstructure transformations on different scales, to capture central aspects efficiently. On the largest scale, we use macroscopic autocatalytic transformation models to effectively describe the transformation behavior, see Section 2.2.1. As discussed above, orientation relationships between the phases play a central role and are discussed in detail in Section 2.2.2.

In the present work, the phase field method is a central anchoring element for the modeling on different scales. The characteristic feature of this diffuse interface approach is to avoid tracking explicitly moving interfaces by introducing a phase field variable, which exhibits spatial variations inside the interface on the scale of the interface thickness. In Section 2.2.3 we discuss the coupling of the various relevant fields in a thermodynamically consistent approach. This discussion is further continued in Section 2.2.4 on the proper relation of phase field models to sharp interface descriptions, which allows to quantitatively simulate situations with finite diffusivity contrast between the phases. For performing phase field simulations, material parameters can nowadays at least partly be obtained using ab initio approaches, which is discussed in Section 2.2.5 for large deformations as required for the press hardening process.

Based on these fundamental modeling ingredients, selected applications are presented in the following sections. On the largest scale of microstructural modeling we pursue a description of bainitic sheaf packets within the aforementioned phase field framework (Section 2.2.6). On the next smaller scale, the evolution of ferritic nuclei is modeled in Section 2.2.7. Thermodynamic and kinetic aspects of the formation of carbides are discussed in Section 2.2.8 and Section 2.2.9, respectively. Finally, the aspect of failure through grain boundary weakening is investigated in Section 2.2.10.

#### 2.2.1. Phenomenological Modeling of Bainitic Transformations

**Figure 9:**
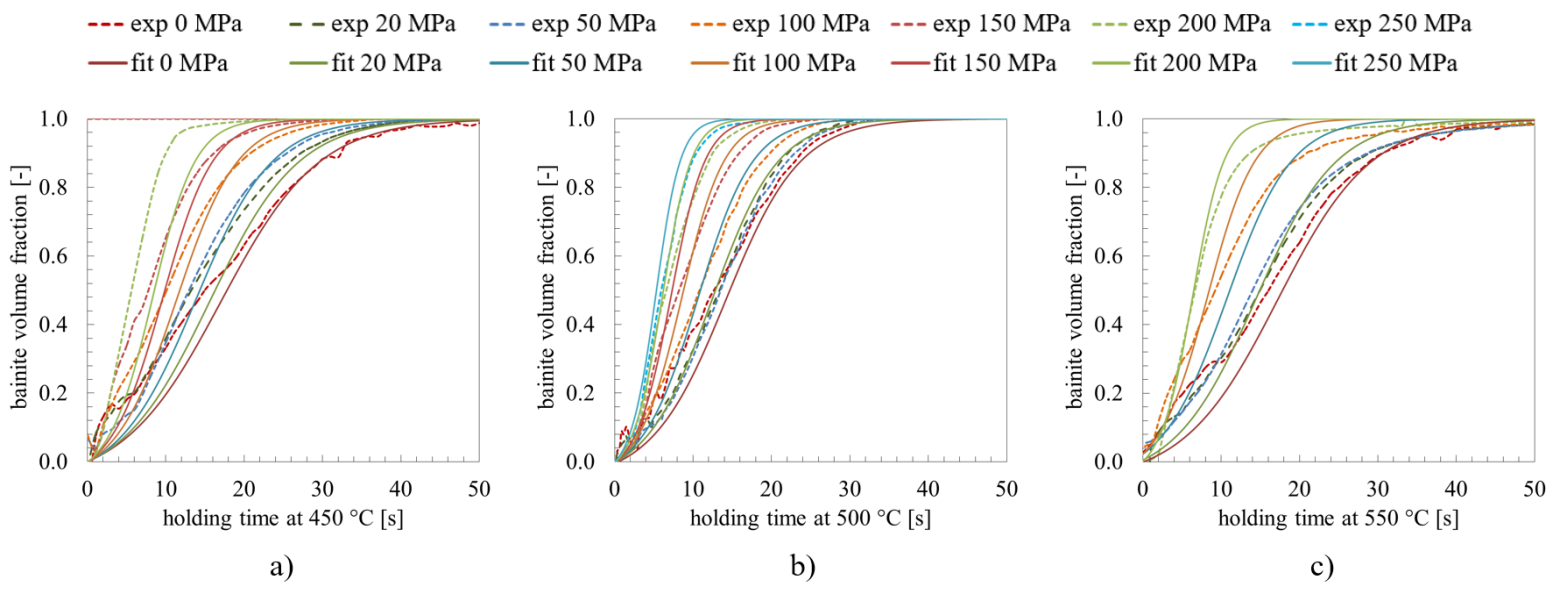
Comparison of simulated and experimental kinetics of the bainitic transformation in regard of applying different stresses for (**a**) 450 ${}^{\circ }C$, (**b**) 500 ${}^{\circ }C$ and (**c**) 550 ${}^{\circ }C$ (adapted from [40]).

Pre-strain and/or high stresses and their interaction are important effects ruling the austenite to bainite transformation that need to be understood and considered for multiscale modeling purposes. Clearly, the press hardening process demands a detailed understanding of the material behavior and the kinetics of the phase transformations. Several central results related to a diffusion based phase transformation approach (autocatalytic transformation model) were published in [34,39,50]. In the following a displacive model is discussed, which combines aspects of several models available in the literature [51,52]. Here, both pre-strain and stress effects are taken into account. The proposed transformation model is given by the following equation:

(1) $$\frac{d\xi_{i}}{dt}=\frac{1}{24}\left(\frac{\Delta G^{\gamma \to \alpha }-U_{i}}{\Delta G^{\gamma \to \alpha }}\right)\frac{kT}{h}\left(1-\xi \right)\left(1+24\lambda \xi_{i}\right)\alpha^{b}\left(B_{s}-T\right)\exp\left(-\frac{Q*}{RT}\right)$$

for the time evolution of fraction $\xi_{i}$ of the i-th variant of bainite. From the 24 variants of an austenite grain, the total bainitic fraction $\xi =\sum_{i=1}^{24}\xi_{i}$ is calculated for this austenite grain. Main parameters are the bainite start temperature $B_{S}$, the activation energy Q*, and a kinetic parameter $\alpha^{b}$. The driving force for the transformation $\Delta G^{\gamma \to \alpha }$ depends on the chemical composition and the stress state. The interaction energy $U_{i}=\epsilon_{i}:\sigma_{i}$ between the lattice deformation $\epsilon_{i}$ and the stress $\sigma_{i}$ has to be calculated for each of the 24 variants. Then, autocatalytic nucleation is considered to happen at the tip of the subunits (causing the needle structure), which is assessed by the autocatalytic factor λ. In the absence of stresses the bias factor plays no role since $U_{i}=0$, therefore the temperature dependent parameters λ, Q* and $\alpha^{b}$ are calibrated under this condition. A further correction of the thermo-chemical driving force allows to reproduce the experimentally observed bainite phase fractions also in the presence of external tensile stresses. Bainite formation is assumed to start with nucleation of subunits due to pre-existing defects at the boundaries of austenite grains, which is captured by the parameter $\alpha^{b}$. The comparison of simulated and experimental kinetics of bainitic transformation for the transformation temperatures 450 ${}^{\circ }C$, 500 ${}^{\circ }C$, and 550 ${}^{\circ }C$ is shown in Figure 9. The applied stress has a much higher influence on the transformation kinetics than the temperature.

A multiscale framework for thermo-mechanical analysis with phase transformations is subject of the published work in [53]. The coupled process at both micro- and macroscopic scales, averaging criteria, mechanical, thermal and phase change constitutive expressions, as well as the corresponding homogenization rules, are derived and discussed in detail in this work.

#### 2.2.2. Bainite Crystallography and Variant Selection

While there is no doubt that the bainitic phase transition itself is displacive, it is still controversial and difficult how a quantitative description of the associated lattice distortion should be formulated [54]. A displacive transformation is accomplished by the cooperative movement of atoms at the substitutional lattice sites, during which the relative displacement of adjacent atoms should be smaller than the interatomic spacing. In the finite strain regime, the lattice distortion strain can be defined by a deformation gradient tensor $F_{ij}=\partial x_{i}/\partial X_{j}$, where *X* and *x* denote the coordinate of atoms prior and after the transformation, respectively. The deformation gradient for lattice distortion $F_{\mathrm{lat}}$ must be derived from crystallographic measurements with various postulations [55,56]. Bain originally described the FCC-BCC transformation as an upsetting deformation [57], which can be expressed in the crystal coordinates system by

(2) $$B=\frac{a_{B}}{a_{F}}\left[\begin{array}{ccc} 1 & \; & \;\\ \; & \sqrt{2} & \;\\ \; & \; & \sqrt{2} \end{array}\right].$$

It gives around 20% strain (positive and negative) in all three principal directions of the unit cell. Later, in the phenomenological theory of martensite crystallography [58,59,60,61,62], the total deformation $F_{\mathrm{lat}}$ is factorised into a rigid body rotation $R_{\mathrm{OR}}$, the Bain strain and a lattice invariant shear strain. An important hypothesis of the phenomenological theory of martensite crystallography and its variants is that $F_{\mathrm{lat}}$ should render a plane invariant and, thus, should take the form of an invariant plane strain,

(3) $$F_{\mathrm{lat}}=R_{\mathrm{OR}}BF_{\mathrm{LIS}}=U+\gamma_{s}\vec{s}⊗\vec{m},$$

where $\vec{m}$ is the unit norm of the habit plane, $\vec{s}$ is the unit vector along the nearly shear direction (due to volume change this invariant plane strain is not a pure shear) and $\gamma_{s}$ is the magnitude of this strain. The assumption of the deformation being an exact invariant plane strain would require that U=I. However, this restriction can be relaxed in some theories, so we denote it as U≊I to keep it more general. The deformation can also render some planes untilted, and the corresponding norm vectors can be calculated as eigenvectors of the matrix $F_{\mathrm{lat}}^{T}$. Different assumptions on the lattice invariant shear systems (dislocation slip or twinning) are required to explain the experimentally observed orientation relationship and habit plane. Despite the large scattering of orientation relationships in various bainite and martensite, one close-packed plane of austenite ${\left(111\right)}_{\gamma }$ is normally oriented within a few degrees to one close-packed plane of the child ferritic grain (011)α. The habit plane of low carbon martensite is also close to this common close-packed plane ${\left(111\right)}_{\gamma }\|\left(011\right)\alpha$.

**Figure 10:**
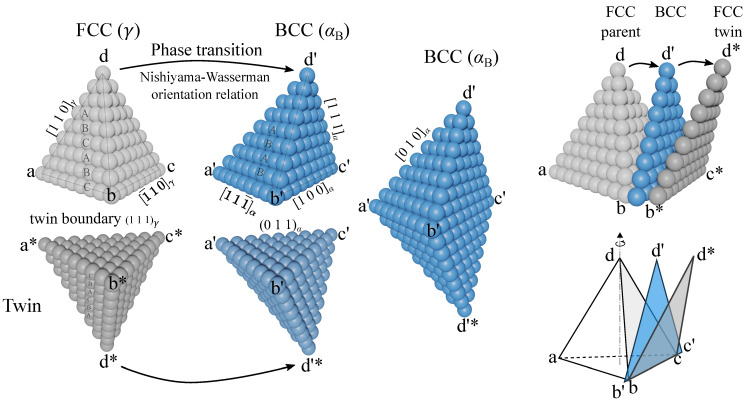
Double Thompson’s tetrahedrons showing the change of atom lattice structure during bainitic/martensitic transformations; The FCC→BCC transformation is represented by the grey and blue tetrahedrons without loss of generality. In this configuration two twin-related FCC lattice tetrahedrons transform into the same BCC lattice if their habit plane ${\left(111\right)}_{\gamma }\|\left(011\right)\alpha$ is coplanar to the twin boundary. Atom correspondence (displacive strain) is illustrated on the highlighted close-packed plane $\bar{bcd}$, on which the phase transition moves the top atom from point *d* to $d^{\prime }$, while twinning moves it to point $d^{*}$. All lattices are drawn with parameters of iron at 420 ${}^{\circ }C$ and the Nishiyama-Wassermann orientation relationship.

The first two parts of the factorized strain, the rigid body rotation and Bain strain $R_{\mathrm{OR}}B$, render a stress-free BCC lattice, which is illustrated in Figure 10 on a Thompson’s tetrahedron (formed by the 4 close-packed planes of the FCC lattice). The austenite crystal is colored in grey and the bainite/martensite in blue. The Nishiyama-Wasserman orientation relationship and the inter-atomic distances for austenite and ferrite at 420 ${}^{\circ }C$ are used to draw the lattices. Applying the 24 rotations $R_{\mathrm{sym}\;}$ within the cubic crystallographic point group *O* (Schoenflies notation) of the FCC lattice renders different child BCC lattices, that are known as orientational variants. The rotated tensor of deformation gradient can be expanded to

(4) $${F_{\mathrm{lat}}}_{\;i}=R_{\mathrm{sym}\;i}UR_{\mathrm{sym}\;i}^{T}+\gamma_{s}\left(R_{\mathrm{sym}\;i}\;\vec{s}\right)⊗\left(R_{\mathrm{sym}\;i}\;\vec{m}\right)\;\mathrm{for}\;i\in \left\{1\cdots 24\right\}.$$

In Figure 10 we neglect the lattice invariant shear and choose the unrotated ${\left(111\right)}_{\gamma }$ plane as the habit plane $\vec{m}$. The shear direction $\vec{s}$ is then $\vec{dd^{\prime }}$, approximately parallel to the ${\left[11\bar{2}\right]}_{\gamma }$ direction. Due to symmetry, the shear plane of a variant $R_{\mathrm{sym}\;i}\;\vec{m}$ must fall into one of the close-packed planes {111} of the FCC lattice.

Variants that share the same shear plane can be grouped together, namely as a close-packed group. Since there are 24 variants and 4 close-packed planes in FCC, there are maximum 6 different $R_{\mathrm{sym}\;i}\;\vec{s}$ for one close-packed group. The shear component of them can cancel each other because of the 3-fold symmetry along the [1 1 1] direction.

**Figure 11:**
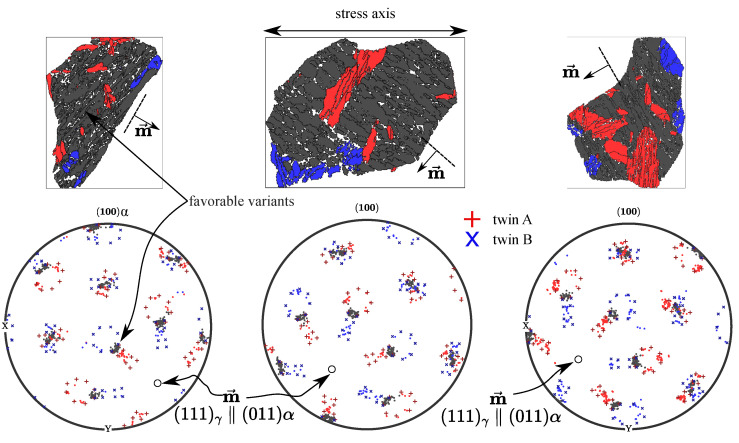
Pole figures for bainitic ferrite sheaves inside individual prior austenite grains; the bainite was formed at 450 ${}^{\circ }C$ under a tensile stress of 50 MPa (Figure 2b). Measured orientations of the sheaves are plotted as fine dots. The calculated variants according to Kurdjumov-Sachs (KS) orientation relationship (OR) are represented by symbols ‘+’ (red) and ‘x’ (blue). The two different symbols distinguish the two twin-related parental austenite orientations. Variants that satisfy the KS OR with only one side of the twins are marked in red or blue, according to their parental orientation. Those six “special” variants that satisfy the OR with both sides of the twins are marked in grey. They share a common close-packed plane that is also parallel to the ${\left(111\right)}_{\gamma }$ twinning plane.

Experimentally, bainitic sheaves of the same close-packed group are typically found to be parallel to each other, forming a set of lamellar grains, namely, a packet. Our EBSD maps show that externally applied stress can significantly increase the size of these packets and reduce the total number of packets formed inside an austenite grain (Figure 2 and Figure 11). It is demonstrated in Figure 10 that two twin-related austenite grains can be transformed into the same ferrite crystal (grains of the same orientation). For such orientational variants, the common close-packed plane ( $R_{\mathrm{sym}\;i}\;\vec{m}⊥\bar{a^{\prime }b^{\prime }c^{\prime }}$) is coplanar to the twin boundary (symmetric plane $\bar{abc}$), and their shear directions $\vec{dd^{\prime }}$ are mirror-symmetric about this plane. This special property is not a coincidence of the orientation relationship, but a natural outcome of the symmetry of the space group. As the stacking sequence of close-packed planes is ABCABC in the FCC lattice and ABAB in the BCC lattice, a mirror operation about their common close-packed plane flips the FCC stacking sequence but leaves the BCC one unchanged. In Figure 11, the poles of bainitic ferrite grains forming from a common parental austenite grain are shown together.

In a (100) pole figure, we can observe two distinct orientation relation patterns (separately marked in red and blue), and the misorientation between them is 60${}^{\circ }$${\langle 111\rangle }_{\gamma }$. Thus, these bainitic ferrite grains must come from two twin-related parental-austenite orientations. The aforementioned “special” orientational variants, whose common close-packed plane is parallel to the twin boundary, can come from both twins. By definition, these special orientational variants belong to the same close-packed group; thus, they can also form a single packet. The bainitic ferrite grains of these special orientational variants are marked in grey color in both the pole figures and the EBSD maps. The other bainitic ferrite grains can only be formed from one side of the twins and they are distinguished by the colors red and blue. It is apparent that this close-packed group (packet) is the dominant one, and its common close-packed plane forms roughly a 45${}^{\circ }$ angle to the axis of externally applied tensile stress, because the resolved shear stress is maximum at this angle. According to Equation (4), the orientational variants of this close-packed group are selected by the tensile stress.

The lattice invariant shear strain $F_{\mathrm{LIS}}$ is attributed to one or more plastic deformation systems (slip and twinning) in different crystallographic models. The purpose of introducing this homogenous strain is to keep a crystallographic plane untilted and undistorted after the total lattice distortion. This plane is the natural habit plane $\vec{m}$ on which the phase boundary can propagate without inducing a long range stress field. However, there is no universally accepted way to measure or calculate the strain tensor related to lattice invariant shear, so it may serve as a fitting parameter in many crystallographic models with various assumptions on the shear plane and direction. A “single shear” can lead to the ${\left\{259\right\}}_{\gamma }$ habit plane in Fe-Ni martensite [58,59,60] and “double shear” can successfully predict the ${\left\{557\right\}}_{\gamma }$ habit plane in low carbon lath martensite [63]. For the ${\left\{225\right\}}_{\gamma }$ habit plane in high carbon steels, there exist many potential models, involving assumptions of dilatation or nano-twins [54,55,62]. Here, we try to avoid imposing such assumptions and consider lattice invariant shear as a general plastic relaxation process that can be modeled by crystal plasticity in a full field model coupled with phase fields.

#### 2.2.3. Full Field Description of Entropy, Free Energy and Dissipation

In various mean field models of the bainitic transformation [27,28,64], the nucleation and growth kinetics are expressed as functions of thermodynamic quantities of the austenite, ferrite and carbide phases, which are typically determined from CALPHAD databases where the influence of stress or strain is not considered. An essential part of thermodynamically consistent chemo-mechanical coupling lies in a correct description of such thermodynamic quantities in given stress-, strain-, composition- and phase fields.

We extended Levitas’ phase field description for martensitic transformation [65,66] in the more general frameworks of Gurtin [67] and Miehe [68,69] using the finite strain scheme. For an arbitrary domain in the deformed (current) configuration $P_{d}$, and in its reference configuration $P_{0}$, three thermo-mechanical-flux metrics are defined on the boundaries ($\partial P_{d}$ and $\partial P_{0}$): traction t=σda=PdA; heat flux qda=QdA; and mass fluxes $h_{i}da=H_{i}dA$. The vectors da and dA denote, respectively, infinitesimal facets on the domain boundaries $\partial P_{d}$ and $\partial P_{0}$. Apparently, σ is the Cauchy stress tensor in the deformed configuration and *P* is the first Piola-Kirchhof stress in the reference configuration. The other metrics are defined comparably in both configurations, between which the geometric transformation can be derived $da=\det\left[F\right]F^{-T}dA$.

Internal energy can then be defined as a functional of the concentration field of solid-solution elements $\left\{c_{i}\right\}$, temperature field θ and the phase fields $\left\{\eta_{k}\right\}$. The changing rate of internal energy is governed by the conservation law,

(5) $$\int_{P_{0}}\dot{e}\;dV=\int_{\partial P_{0}}\left(-Q+\dot{x}P-\mu_{i}H_{i}\right)\;\cdot \;\;dA.$$

The Einstein summation notation is used here, so the repeated indices are summed over. The scalar μ denotes the chemical potential, the energy conjugate of the mass flux by diffusion. Neglecting body forces, momentum and external mass supply, we can apply Gauss’ theorem to get the expression of the changing rate of internal energy density,

(6) $$\dot{e}=-\mathrm{div}Q+P:{\dot{F}}^{T}-\nabla \mu_{i}\;\cdot \;H_{i}+\mu_{i}{\dot{c}}_{i}.$$

Dissipation can be defined as the sum of entropy production $\dot{s}$ in the system and entropy output to the environment,

(7) $$\int_{P_{0}}\frac{D}{\theta }\;dV=\int_{P_{0}}\dot{s}\;dV+\int_{\partial P_{0}}\frac{Q}{\theta }\;dA.$$

A Legendre transformation of the internal energy density *e* yields the Helmholtz free energy density ψ:=e−sθ. Then, the dissipation energy density can be expressed as

(8) $$D=P:{\dot{F}}^{T}+\left(-\dot{\psi }-s\dot{\theta }+\mu_{i}{\dot{c}}_{i}\right)-\nabla \mu_{i}\;\cdot \;H_{i}-\nabla \theta \;\cdot \;\frac{Q}{\theta },$$

where the first term is the stress work and the second term (in brackets) is the partial free energy reduction. The third and fourth ones are, respectively, the local dissipation by solid-state diffusion and heat transfer. The decomposition of the deformation gradient into elastic and inelastic parts, $F=F_{e}\;\cdot \;F_{\mathrm{ie}}$, is used to separate the reversible and irreversible parts in the stress work,

(9) $$P:{\dot{F}}^{T}=\left(F_{\mathrm{ie}}P^{T}\right):{\dot{F}}_{e}+\left(P^{T}F_{e}\right):{\dot{F}}_{\mathrm{ie}}\;,$$

where the second term is related to plastic relaxation (slip or twinning), transformation induced lattice distortion, excess volume of solid solution and thermal expansion.

In a phase field model, we introduce order parameters $\left\{\eta_{k}:k=1,2,\cdots ,N\right\}$ to describe the existence of bainitic ferrite grains and their gradients $\left\{\nabla \eta_{k}\right\}$ to describe boundaries. The orientations of bainitic ferrite grains are assigned according to the orientation of the parent austenite grain and the rotation matrix for one of the orientation relation variants. The eigenstrain or stress-free transformation strain for each grain is given by Equation (4). Therefore, both the Helmholtz free energy density field ψ and the inelastic strain field $F_{\mathrm{ie}}$ can be formulated as functional of $\left\{\eta_{k},\nabla \eta_{k}\right\}$, from which the kinetic equations for all fields can be derived.

#### 2.2.4. Non-Diagonal Phase Field Model and Thin Interface Limit

To simulate extended systems and reduce the computational effort of phase field descriptions, the numerical interface width is often taken significantly larger than the true physical width of an interface, which is typically of the order of a few atomic distances. However, this increase by several orders of magnitude induces undesired interface effects.

In [70], an asymptotic analysis linking the classical phase field model and the corresponding sharp interface description was introduced and called the thin-interface limit for the case of equal diffusivities in the parent and growing phases (known as symmetric model). Later, the thin-interface limit was extended to the case of alloys with a negligible diffusive transport in the growing phase (known as one-sided model) through introducing an extra term known as anti-trapping current. However, for solid-solid transformations, a finite diffusivity contrast between the growing phase and parent phase is a characteristic feature, contrary to the aforementioned limiting cases of symmetric and one-sided transformations. In this situation, the thin-interface analysis of the classical phase field model leads to the conclusion that proper equilibrium boundary conditions cannot be obtained without modification of the interface thermodynamics using unphysical adsorption. Recently, this limitation has been overcome by a new kinetic cross-coupling term between the phase field and the diffusion field, in a way that it obeys the Onsager reciprocity relations. In the constitutive force-flux relations of the phase field model, the cross-coupling term provides an additional degree of freedom and the anti-trapping current naturally appears to achieve the correct equilibrium boundary conditions at the moving interface. The heat exchange for a small temperature difference between the transformation front and the environment has been modeled in one dimension first, in order to validate the non-diagonal phase field model without consideration of surface diffusion. Nevertheless, surface diffusion, i.e., a tangential flux driven by gradients in the chemical potential along the interface, takes place inside the phase field interface layer, and is typically artificially enhanced compared to real physical systems. Therefore, it is essential to tune the surface diffusion to get the proper interface dissipation in the non-diagonal phase field model. This extension has been made in [71], and consequently the non-diagonal phase field model reproduces well the free boundary description where both the Kapitza jump and the (artificial) surface diffusion are eliminated, allowing for quantitative phase field simulations with a broad range of diffusivity ratios between the phases.

A benchmarking of the model against sharp interface Green’s function approaches is discussed in Section 2.3.1.

#### 2.2.5. Ab Initio Parameter Generation

An essential ingredient for the scale bridging modeling of bainite is the computation of material properties. Elastic parameters are of central relevance for the press hardening process due to the strong interplay between chemical, thermal and mechanical degrees of freedom. Nowadays, ab initio approaches allow to predict elastic constants with rather high accuracy for low temperatures. For elevated temperatures, the descriptions can be extended using quasiharmonic approximations, which take phonon excitations into account. However, these descriptions reflect only the linear deformation regime, and for high strength materials at least locally high strains and stresses can play a role. A quantitative prediction of the elastic nonlinear response can however easily become complex due to the high number of emerging parameters in the different phases.

In [72] we developed a structural understanding of large elastic deformations, which results in closed analytical expressions for direct use in larger scale models. Starting point is the generation of a large set of ab initio data, here especially for bcc materials, for different mechanical loadings. In particular for hydrostatic loading, the Birch-Murnaghan curve is frequently used in ab initio calculations to obtain a quantitative fitting of energy-volume curves,

(10) $$E\left(V\right)=E_{0}+\frac{9V_{0}K}{16}\left\{{\left[{\left(\frac{V_{0}}{V}\right)}^{2/3}-1\right]}^{3}K^{\prime }+{\left[{\left(\frac{V_{0}}{V}\right)}^{2/3}-1\right]}^{2}\left[6-4{\left(\frac{V_{0}}{V}\right)}^{2/3}\right]\right\}.$$

Here, $V_{0}$ and *V* are the equilibrium and actual volume for an isotropic deformation, respectively. *K* the bulk modulus at zero pressure and $K^{\prime }={\left(dK/dP\right)}_{P=0}$ the derivative of the bulk modulus with respect to pressure. This rather lengthy expression corresponds to a Langrangian description of the deformation state relative to a non-deformed reference state. In a Eulerian description, this expression becomes significantly simpler and reads

(11) $$E\left(V\right)=\frac{9}{2}KV_{0}e_{xx}^{2}\left[1+\left(4-K^{\prime }\right)e_{xx}\right],$$

where the Eulerian strain is $e_{xx}=\left(a-a_{0}\right)/a$, using the undeformed and deformed lattice constants $a_{0}$ and *a*, respectively. The expression (11) shows clearly the meaning of $K^{\prime }$ as a higher order correction of the linear elastic energy, corresponding to a nonlinear stress-strain relationship. The decisive step is the linking of this description to phase field crystal and classical density functional formulations of bcc materials. We have elaborated amplitude equations descriptions, which naturally contain elastic deformations, and which are similar to conventional phase field descriptions but encode in the complex phase of the order parameters (compared to real-valued phase field descriptions) the local state of the elastic deformation, as well as extended defects like dislocations and grain boundaries [73]. It turns out that the rotation invariant differential operator in the generating free energy functional is responsible for the same type of nonlinear elastic response, as described by the Birch-Murnaghan equation and the ab initio simulations. Moreover, we obtained $K^{\prime }=4$ from this description, and this value is in convincing agreement with the ab initio results. Therefore, the obtained nonlinear stress-strain relations, also beyond hydrostatic deformations, as illustrated above, provide closed form expressions for nonlinear elastic deformations in the low temperature regime, which can directly be used in finite element analysis or phase field modeling.

#### 2.2.6. Phenomenological Phase Field Description of Bainitic Sheaf Packets

**Figure 12:**
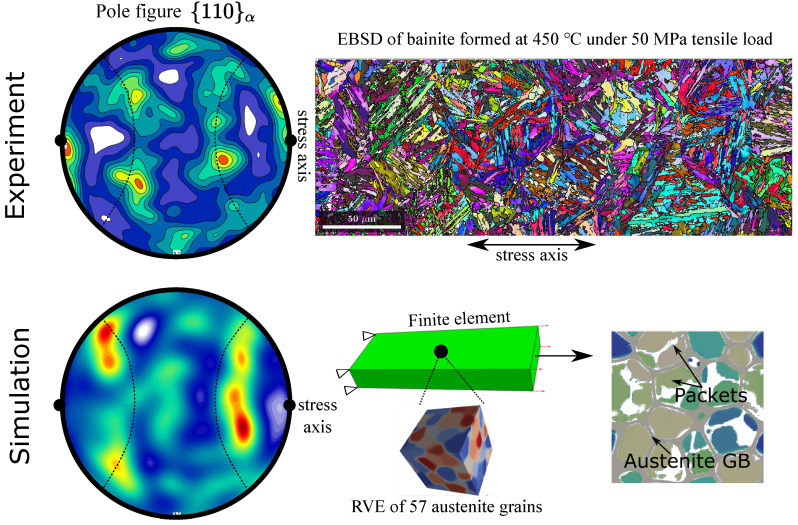
Simulated bainitic microstructure and texture with comparison to the experimental results; favorable alignment of the close-packed planes of bainitic ferrite grains can be observed in the pole figures.

As a first application of the aforementioned model developments we discuss the transformation kinetics on the level of bainitic sheaf packets using a phase field description. For that, a considered volume element of the bainitic microstructure should comprise a large number of prior austenite grains to be statistically representative. At this length scale, a coarse grained representation of the bainitic ferrite sheaves is necessary for the balance between representative volume element (RVE) size and computational costs of the model. Therefore, in [74,75,76], we have described every bainitic packet (sheaves of the same close-packed group) by individual order parameters. The mechanical and chemical driving forces are derived as variational derivative of the free energy functional. The mechanical driving force $\left(P^{T}F_{e}\right):{\tilde{F}}_{\mathrm{lat}\;k}$ depends on the orientation of the common close-packed plane of the packet. We assume that nucleation occurs only at the austenite-austenite grain boundary, and the energy barrier for this process is approximated as the product of the total driving force and the critical nucleus volume. However, we want to point out that there is no clear way to determine the shear component of ${\tilde{F}}_{\mathrm{lat}\;k}$ for each packet, and also the gradient of order parameters does not represent physical boundaries; thus, this approach is phenomenological. Using the multi-phase-field framework of Steinbach et al. [77], different interfacial properties can be assigned to the austenite-austenite grain boundaries, austenite-packet boundaries and the inter-packet boundaries. In [74,76], phase field simulations have been coupled with a finite element simulation on the macroscopic scale. For each integration point of the finite elements, an individual phase field simulation domain is created, where the stress tensor of the integration point is incrementally applied to the phase field simulation. A three-dimensional representative volume element of 60–70 equiaxed austenite grains is used for each integration point. The austenite grain structure is firstly generated using a Voronoi tessellation; then, a short grain-growth step is performed to relax the austenite grain-boundary structure. After the grain growth stage the temperature starts to drop, and the chemical driving force increases as a result, triggering the nucleation and growth of bainitic packets of different orientational variants. The homogenous transformation induced strain for the phase field domain is calculated from the volume fractions of all variants of all austenite grains. The strain tensor is then incrementally transferred back to the finite element integration point where the macroscopic mechanical balance is solved [39,50]. Figure 12 shows the simulation results of bainitic transformation under a tensile stress of 50 MPa with comparison to the experimental results calculated from the EBSD measurements in Section 2.1. The simulation can reproduce the overall transformation induced strain and the effect of variant selection on bainitic packets. For further details of the simulation approach and results, we refer to [74].

#### 2.2.7. Phase Field Model of Single Bainitic Ferrite Nuclei with Crystal Plasticity Relaxation

**Figure 13:**
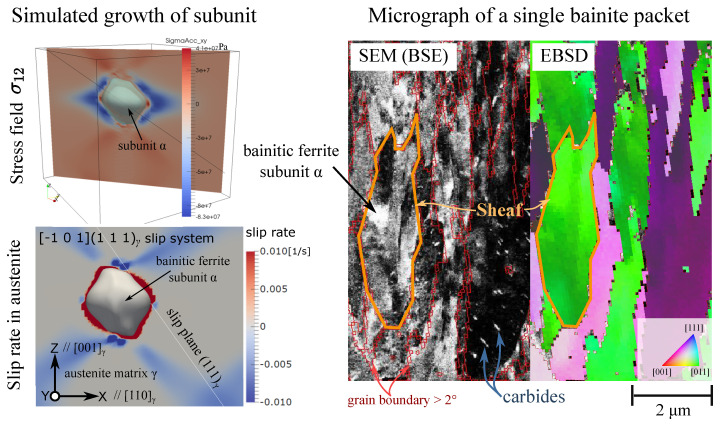
Growth of a bainitic ferrite subunit in the austenite matrix (**left**) and traces of subunits in SEM micrographs (**right**).

At a lower length scale, a physics based description of relevant fields during the bainitic transition can be achieved. In [75], we have introduced a coupled phase-field and crystal-plasticity model, where the dissipative process around a growing bainitic ferrite grain in the matrix of an austenite grain is simulated by the constitutive equations of crystal plasticity implemented in the open-source Düsseldorf Advanced Material Simulation Kit (DAMASK) [78]. Here, the lattice distortion matrix $F_{\mathrm{lat}}$ from crystallographic theory is used (Equations (3) and (4)). As mentioned before, we avoid imposing any invariant plane strain tensor ($F_{\mathrm{LIS}}=I$), because the dislocation slip is already modelled in DAMASK. The bainitic ferrite nuclei is placed as a sphere at the center of the domain, and its isothermal growth is simulated. Isotropic interfacial energy and mobility are used in this model, so that the only anisotropic property of each phase is the definition of the slip systems. The bainitic ferrite grain evolves to a disk shape and the habit plane orients at a small angle to the common close-packed plane (Figure 13). With the full field description at this scale, we can use Equation (8) to calculate the dissipation energy induced by slip as a result of plastic accommodation of the lattice distortion. The dissipation from phase boundary migration and diffusion can also be calculated with the evolution history of the corresponding fields. The volume averaged dissipation energy can then be used in an upper-scale model to estimate the basic thermodynamic and kinetics parameters.

We have also discussed the elastically dominated regime in diffusion-limited solid state transformations in [79], as relevant for the initial and terminal stage of subunit growth into the retained austenite during bainitic transformations. In the early regime related to nucleation, the bulk crystal structure is typically not yet developed to a full extent inside a nucleus, and therefore this phase appears to be softer from an elastic perspective. We find for the assumptions of elastic softening and effective isotropy of the onsetting phase that the problem can be considered analogous to a crack formation problem. When the Mullins-Sekerka instability triggers the transition from this early, elastically governed growth regime to the coupled diffusive-displacive growth regime, the transport of carbon in the retained austenite is assumed to be the limiting factor of subunit growth. Taking into account the decrease of the carbon diffusion coefficient with decreasing temperature, we have studied a zero velocity limit of the transformation in consideration of an elastic frustration, i.e., reduction of the effective driving force, as detailed in [79].

#### 2.2.8. Carbide Formation: Thermodynamics

The formation of carbide precipitates is central for the bainitic transformation, and the driving forces for this process is a reduction of the Gibbs energy by phase separation compared to single phase situations as a complex interplay between thermodynamic and kinetic aspects. Carbide precipitation can take place both in the austenite and bainitic ferrite, where the latter has a low carbon solubility limit. The carbides form preferentially on one (upper bainite) or the both sides (lower bainite) of interfaces between these two phases.

To separate thermodynamic and kinetic effects, we have investigated the striking proximity of carbides near different types of interfaces from a thermodynamic perspective, with emphasis on elastic effects, which can cause a long-ranged interaction between precipitates and the interfaces. An important conclusion is, that this interaction has consequences also for the thermodynamics of phase coexistence, in particular solubility limits (e.g., for carbon), and hence we obtain locally modified phase diagrams. Interestingly, these thermodynamic effects appear as bulk rather than interfacial effects, since elastic deformation are a volumetric effect.

Based on our earlier work [80], where the elastic interaction between precipitates and mechanically free surfaces was studied, we have generalized the concepts to the formation of secondary phases near grain boundaries in [81]. Starting point is the energetically unfavorable appearance of coherency stresses in two-phase bulk situations [82,83], which leads to a partial suppression of phase separation. For coherent precipitates which form near grain boundaries, stress relaxation via shear coupled motion of the grain boundary becomes possible. This migration mechanism connects a lateral relative motion of two grains with a normal motion of the grain boundary. Naturally, shear coupled motion causes elastic stresses, and therefore a deformation of the grain boundary is energetically unfavorable [84], as well as the formation of coherent carbide precipitate itself. However, the elastic cross term resulting from the interaction of the distortion around a precipitate and the stresses generated by a shear coupled grain boundary can be negative and stronger then the two aforementioned “self energies”. From this, an attractive interaction between grain boundaries and precipitates arises, which favors carbide formation near grain boundaries compared to locations in the bulk. We transferred this information into a thermodynamic framework for the description of the low concentration branch of the solubility limit and found a strong reduction of the carbon solubility limit in the ferrite phase near grain boundaries, with a reduction of around 50% in the room temperature regime [81]. This description delivers detailed information on the interaction and also the equilibrium deformation of the grain boundaries.

For a transfer to larger scale descriptions, which do not require this detailed modeling, an effective approach ascribing reduced elastic constants to a “soft” grain boundary allows to capture this effect without spatially resolving the grain boundary contour, e.g., in simulations of bainitic microstructures [85]. A quantitative matching between the microscopic and mesoscopic descriptions, including a perspective to generalize the approach to precipitation near interfaces between different phases (e.g., carbides near interfaces between ferrite and austenite in bainitic steels) using phase field approaches is discussed in [86].

#### 2.2.9. Carbide Formation: Kinetics

**Figure 14:**
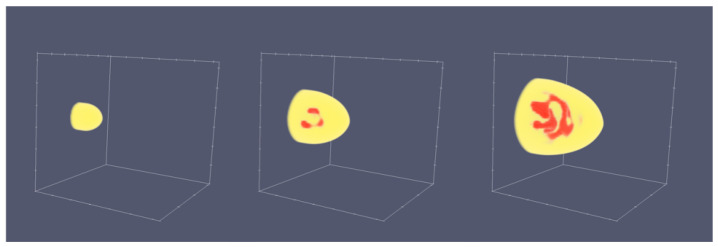
Three-dimensional simulation of the austenite-to-bainite transformation on the subunit level using the phase field model. The surrounding austenite is not shown, the bainitic ferrite is in yellow and the carbides in red, demonstrating their complex microstructure, which results from a chemo-mechanical coupling during the carbide formation via spinodal decomposition.

Based on the above work a phase field model for the bainitic transformation involving a full thermo-chemo-mechanical coupling has been formulated, in the spirit of the concepts in [87,88]. Additional to the approach of Arif et al. [31] we take into account carbides and their microstructural evolution, which is important for a full hierarchical understanding of bainite formation, and in particular the distinction between upper and lower bainite, as well as the consideration of diffusive and displacive transformations. The model extends the previous approaches in the sense that the mechanical problem is solved on the basis of an elastic representation, where stresses are generated by mismatch stresses at coherent interfaces and external stresses. Chemically induced stresses due to spatially varying carbon concentrations are taken from ab initio simulations. With this model we are able to apply stresses through boundary conditions to the simulation, to simulate the press hardening process of bainite on the subunit level. The focus of our investigations is on the microscopic evolution, i.e., the formation of a lath- or needle-like structure of bainite. 22MnB5 is considered to result in the generation of lower bainite [89], featuring the forming of carbides inside the ferritic lath structure. This behaviour strongly depends on the carbon concentration and transformation temperature. The carbon diffusion and segregation is represented in terms of a spinodal decomposition process [87]. For bainite formation precipitation of carbides is possible in the bainitic ferrite if a critical concentration is exceeded. This allows the system to reach a stable carbon concentration in bainite and simultaneously correctly presents the carbides as carbon sink holes [90]. A result of a growing bainite lath is depicted in Figure 14.

A small nucleus of bainite at the start of the simulation grows to a needle-like structure due to the strong anisotropy. Inside the bainite phase the carbon diffusion is active, leading to segregation of carbon. The carbon diffusion in austenite is not considered, as carbides inside the lower bainite are more significantly influencing the microstructure. Altogether, the presence of mechanical effects significantly alters the microstructure morphology.

**Figure 15:**
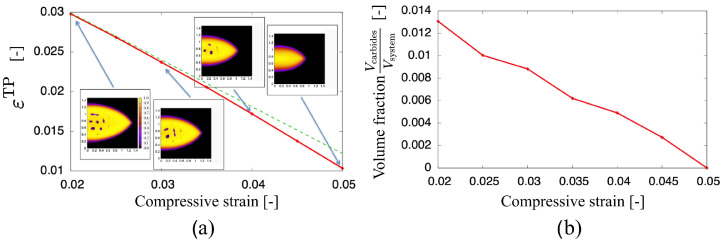
(**a**) Resulting transformation plasticity on the subunit level due to the application of an external compression during the bainitic transformation in a plane strain setup. During the transformation, a perpendicular compressive strain is applied in the direction perpendicular to the bainite front propagation. It is released after the process, and a remaining stress free strain $\epsilon^{\mathrm{TP}}$ is resulting from the phase transformations. The dashed line is a linear fit to the low strain data, emphasizing the nonlinear transformation plasticity at larger applied strains. The model is based here on elasticity and phase transformations only, which is a suitable description in the small strain regime. (**b**) Carbide phase fraction as function of the external compressive strain.

Also, we have inspected the influence of external stresses during press hardening also on the subunit level. A particular question is related to the role of bulk plasticity, phase transformations and elastic contributions, and how they affect anisotropic length changes after the transformation. In agreement with the experimental findings [34] a linear relationship between the transformation plasticity (width strain) and the applied strain is found for low strains, see Figure 15.

Deviations appear at larger strains, which are attributed to the different volume fractions of the carbides. For further details, we refer to [91].

#### 2.2.10. Grain Boundary Failure

During the fabrication process and partially during the austenization step of the press hardening process, the steel is exposed to high temperatures, and then grain boundary induced failure may occur under the additional influence of high mechanical stresses. This effect can be a consequence of grain boundary premelting processes due to the formation of thin disordered layers already significantly below the bulk melting point. Such a failure mode is well known in metallurgical processes as transverse cracking and can be measured by hot ductility tests. In general, the effect is provoked by the interaction of nearby order-disorder interfaces, and can be expressed as an effective interaction between them. This effect has been investigated experimentally and in many theoretical and numerical studies, see [92] for an overview. It turns out that high angle grain boundaries have a tendency for premelting in the aforemented sense, whereas low angle grain boundaries remain unaffected. This effect is most prominent in alloys, for which grain boundary premelting can start at about 60–85% of the bulk melting temperature, and can furthermore drastically be enhanced by mechanical deformations.

We have investigated how grain boundary premelting can be captured in phase field models for microstructure evolution simulations [77], as used in the present paper. In such a description, interactions between solid-melt interfaces arise if the phase field profiles with non-vanishing thickness overlap. For that purpose, the stationary phase field equations for planar interfaces are solved, leading to set of coupled linear differential equations, providing an analytical expression for the grain interaction across the separating premelting layer. The decisive parameter is the difference of interfacial energies for a dry grain boundary situation and a wetting case, where two solid-melt interfaces appear. In case that the dry grain boundaries are energetically less favorable, we get premelting already below the bulk melting temperature and the two appearing solid melt interfaces experience a repulsive interaction. In the opposite case, the grain boundary can stay dry even slightly above the melting point due to an attractive interaction. Beyond these stationary situations we have performed phase field simulations for the propagation of wet layers along grain boundaries and compared the front velocities to sharp interface predictions [92].

### 2.3. Benchmarking and Bainite Toolbox

The development of models for microstructure evolution and property prediction of bainitic steels is a central challenge also beyond the results presented here. For the comparison of existing and new developed descriptions a proper benchmarking is therefore essential, in order to identify strengths and limitations of the approaches. For the outreach of central results we have developed a selection of simulation tools, supplemented them with graphical interfaces and collected them within a “bainite toolbox”. Examples of both benchmark scenarios and software tools are briefly summarized in the following.

#### 2.3.1. Benchmarking

A central part of the model development presented in this paper is dedicated to the development of suitable phase field models to predict the microstructure evolution. To benchmark these models also in a more general context, comparison to dedicated experimental investigations and also analytical modeling is essential. For this reason, a benchmarking of the phase field descriptions against experimental investigations of microstructure evolution during solid state transformations was performed in [93] for lamellar morphologies. A central aspect was the extraction of proper scaling laws for the coarsening of interfacial energy driven phenomena in comparison to existing models in the literature [94,95]. It turns out that the predicted growth experiments in these analytical works do not explain the observed experimental and simulation results, and instead a classical Lifshitz-Skyozov-Wagner exponent of n=1/3 for the lamellar spacing, λ∼$t^{n}$, leads to the most convincing description over a large range of time and length scales in a consistent agreement between experimental investigations and phase field simulations.

As pointed out above, the interaction of composition and stress is a central element of the bainitic microstructure in multiple ways, as well as for other applications. Therefore, a benchmark setup with composition gradients and elastic deformations around a single spherical precipitate particle was simulated with 3 different numerical approaches, where the transformation kinetics and morphological aspects of precipitation are expected to be largely influenced both by the chemical and the mechanical evolution in the system. In this benchmark problem, the elastic energy is a function of chemical composition *c* and order parameter η; consequently, the local chemical potential is influenced by the stress field, causing stress-gradient driven diffusion. We simulate the same problem with 3 numerical implementations involving 2 popular open-source software, DAMASK and OpenPhase [78,96]. OpenPhase has two solvers: the chemical solver for kinetics equations of fields {c,η} and the mechanical solver for static stress equilibrium. We have compared results from the following three numerical combinations: OpenPhase only, DAMASK only and the OpenPhase chemical solver coupled with the DAMASK mechanical solver. All numerical results agree well with each other with a mean deviation of around 5% (for the concentration field including the diffusive interface area) [97].

Based on the development of the nondiagonal phase field model in Section 2.2.4, two-dimensional simulations of solidification of a pure substance with elimination of all the abnormal interface effects have been performed. We point out that the quantitative modeling of dendritic solidification is considered to be a “gold standard” for the qualification of modeling descriptions due to the advanced understanding of this microstructure and the availability of high precision Green’s function descriptions. Therefore, such Green’s function calculations have been performed to benchmark the non-diagonal phase field simulations in the symmetric and one-sided limits. As detailed in [98], the non-diagonal phase field simulations reproduce the Green’s function results in a satisfactory manner with less than 5% deviation. For two-sided cases, the phase field simulations get closer to the analytical theory when the undercooling decreases. Besides, the robustness of the phase field results are supported by two other sets of simulations when (i) the kinetic cross-coupling vanishes, and (ii) the surface diffusion remains. The deviation approaches 20% when the kinetic cross-coupling vanishes, while it reaches 10% when the surface diffusion remains. We refer to [98] for further details of the comparison. The comparison emphasizes the necessity of the necessity of using the non-diagonal phase field model with elimination of surface diffusion. Therefore, the non-diagonal phase field model provides a quantitative basis to reproduce the microstructure evolution process during bainitic transformation with full consideration of the diffusion difference between the austenite and ferrite phases. We note that in contrast to solidification situations, where often the diffusional transport in one phase can be neglected or the transport can assumed to be the same (symmetrical model for thermal dendrites), solid state transformations are often in between these limiting cases. Before, quantitative phase field descriptions with controlled thin interface asymptotics have not been available, and the present extension towards a non-diagonal model allows to capture the unequal diffusional transport during bainitic press hardening processes quantitatively.

#### 2.3.2. Bainitic Micrograph Across the Scales

An important feature of bainitic microstructures is their multiscale nature. Therefore, bainitic micrographs need to be investigated and presented on the whole 10 nm to 100 μm range with multiple layers of data acquired from different detectors. This typically implies that the covered region of interest should comprise enough number of prior austenite grains for a statistical representativeness of the whole microstructure. For this purpose, we use leaflet, a popular open-sourced tool for displaying street maps and satellite images, to facilitate the interactive exploration of the bainitic microstructure across the scales. Multiple high resolution SEM images are spatially aligned with each other and placed in a global coordinate system. Signals from different SEM detectors (secondary electron, backscattered electron and EBSD pattern) reveal different features of the microstructure, so they should be spatially registered and mapped onto each other. Derived features, such as prior austenite grain boundaries and orientations, are contained in the micrograph to help understanding the relation among different phases and grains. A press-hardened bainitic microstructure of 22MnB5 formed in a mold of 420 ${}^{\circ }C$ is openly accessible at the webpage [99]. The entire micrograph is compiled from 9 high-resolution SEM backscattered electron images, where carbides are visible at the highest zoom level. More details of carbides in this microstructure can be found in Figure 1 of reference [86]. A layer of EBSD map (kernel average misorientation color coded) is superimposed on top of the SEM image. A small region of the micrograph that covers a single packet is shown in the right side of Figure 13.

#### 2.3.3. Precipitate Induced Grain Boundary Deflection

As mentioned in Section 2.2.8 the strong chemo-mechanical coupling between precipitates and interfaces, in particular grain boundaries, can lead to significant changes of the apparent equilibrium solubility limits e.g., for carbon. For a visualization of the deflection of the grain boundary, induced by the elastic interaction with a nearby array of precipitates, we generated a Java based application. There, the geometrical and material parameters can be adjusted, and the predicted influence on the grain boundaries is visualized.

#### 2.3.4. Machine Learning Predictions of the Bainite Start Temperature

For the control of the bainitic microstructure, the knowledge of the bainite start temperature is important. The exact value of this temperature is sensitive to steel alloying and depends sensitively on other conditions, hence even a qualitative understanding is still challenging. Theoretical predictions based on experimental observations are still controversial and related to the debate of the displacive-diffusive transformation [22,100,101]. For both perspectives transformation kinetic models have been developed [23,24,51,102,103]; in this way, predictions for the transformation temperature in the sense of linear or multilinear regressions in combination with empirical models have been elaborated [104,105,106]. This class of approaches offers the possibility of very good transfer and interpretation, but is limited in its range of validity. Complementary, thermodynamic calculation frameworks [107] have the ability for higher accuracy of the bainite start temperature prediction by consideration of thermodynamic criteria [108,109].

For a further improvement of the predictions, we have developed a novel data augmentation concept for the prediction of the bainite start temperature. It links thermodynamic principles and databases with artificial intelligence approaches. For this purpose, we use training and testing data which is taken from published and validated experimental measurements, which contains in particular variations of the alloy composition and resulting measured start temperatures. The conceptual idea is here to supplement the data by the carbon chemical potential in ferrite, evaluated at a guessed bainite start temperature. To test this augmentation approach, we have first calculated the accuracy of the predictions using an artificial neural network which operates on the original data set only. These results are compared to the augmentation approaches which additionally use the chemical potential at the experimentally determined bainite start temperature, and alternatively for two approximated values for this transition temperature. We point out that the use of the measured bainite start temperature is less useful for practical applications, as the prediction of the transition temperature is the goal and not available a priori; nevertheless, this approach is useful to estimate the theoretical accuracy limits and the information gain by the augmentation approach. We find that the different approximation schemes are indeed suitable to predict the bainite start temperature as function of composition. Depending on the necessary computational effort, we can reach a mean absolute error of about 14 or 4 Kelvin for the different approaches, which is in the same confidence range as the experimental measurements [110]. The trained artificial neural networks are implemented as a standalone tool as part of the bainite toolbox.

## 3. Discussion

In this paper, we have summarized our recent progress in a combined experimental-modeling understanding of bainitic transformations under the influence of mechanical stress, as relevant in particular for the press hardening process.

A key step for the development of new models is an understanding of the crystallographic aspects of the transformation between austenite and bainite. In all existing models for bainitic and martensitic transformations, BCC grains of maximum 24 orientations are expected to form within a single austenite parental crystal. Experimentally, we found a significant amount of austenite twins. We have shown in Section 2.2.2 that twinning in the austenite lattice followed by the rotation matrix of a normal orientation relationship can better explain the observed orientation distribution: that is, up to 42 distinguishable orientations can form inside a prior-austenite grain with a pair of twins. This twinning occurs most probably along the close-packed plane with highest resolved shear stress, which also serves as the common close-packed plane of the favorable orientation variants. These findings explain the alignment of bainitic needles at 45${}^{\circ }$ to the stress axis reported in previous studies on various steels [5,9,10]. It is not clear whether the twinning of austenite occurs prior to the bainitic transformation or simultaneously with the phase transition. Although the externally applied stress is lower than the yield stress of austenite, mechanical twinning can be activated by the internal stress field of previously formed bainite sheaves in the vicinity. In this case twinning of austenite is a mechanism for self-accommodation of the lattice distortion strain. In the original phenomenological theory of martensite crystallography [58,59,60] and more recent crystallographic theories for martensite [55,111], twinning in the BCC phase is an important mechanism of lattice invariant shear (in Equation (3)), where the combination of a pair of twin-related martensite laths can render an invariant plane strain with a habit plane of ${\left\{259\right\}}_{\gamma }$, ${\left\{557\right\}}_{\gamma }$ or ${\left\{225\right\}}_{\gamma }$, observed in major kinds of martensite. For bainite, the widely existing twin-related austenite might indicate a different transformation mechanism to martensite.

The understanding of carbide formation in the proximity of these interfaces is important in particular for the understanding of the distinction between upper and lower bainite. We have demonstrated that the carbide formation is strongly affected by thermodynamic effects, which are effectively locally altered near surfaces, grain boundaries and interfaces due to elastic precipitate-interface interactions. Typically, this leads to a stronger tendency for carbide precipitation near such interfaces. Kinetically, we have demonstrated in phase simulations that elastic and plastic effects substantially affect carbide formation in a sense of a strong thermo-chemical coupling. Consequently, externally applied mechanical loadings during the press hardening process affect the amount and orientation of carbides.

On the methodological side, the modeling of complex hierarchical microstructures in bainite requires advanced models, which rely on a large number of ingredients and their proper interplay with each other. The strong thermo-chemo-coupling during press hardening implies that thermodynamic, kinetic, chemical and mechanical effects contribute to the evolution and finally to the material properties with similar weights, and hence driving forces e.g., in phase field simulations cannot be separated into dominant and subdominant contributions. For a deeper understanding and to ensure model correctness, it is therefore essential to investigate well controlled benchmark situations and to compare predictions to complementary approaches with rely on alternative numerical concepts but the same underlying physical models. In this way, a controlled development of models with the ability to predict confidence intervals becomes possible, and a detailed investigation of modular exchanges, e.g., between elastic and plastic coupling, becomes feasible.

Here, the progress in phase field modeling approaches over the past decades in combination with increasing computational power nowadays allows to perform complex simulations with hierarchical microstructures under the consideration of multiple ingredients, in particular elastic and plastic deformations. The advantage of variational phase field models is that they allow to incorporate additional effects by inclusion in generating thermodynamic functionals in a thermodynamically consistent way. Moreover, a next generation of phase field models extends the descriptions to nondiagonal models, which allow for a precise control of the interface kinetics also in situations, where diffusive transport plays unequal roles in adjacent phases. This is of particular interest for solid state transformations like bainite formation, where different but finite carbon diffusivities are essential for the microstructure formation.

Furthermore, we expect that the recent advances in machine learning techniques will also have increasing impact on the understanding of bainitic microstructures. Due to the complexity of the problem, direct correlations between modifications of composition and processing conditions are often difficult to identify directly, and therefore the computed guided search for material optimizations is a promising supporting tool. We note that typically the available data sets are sparse due to the high dimensional configuration space, and additionally they are often defective, which requires the development of suitable imputation schemes.

## 4. Conclusions

Today, the bainitic microstructure is to a large extent still insufficiently understood, which is mainly due to its multiscale and nonequilibrium character, in combination with competing mechanisms and their complex interplay. The application of external stresses, in addition to internal stresses which arise due to phase transformations and coexistence, adds a new degree of complexity due to the strong thermo-chemo-mechanical coupling. In this paper we have summarized our recent ambitions to provide novel modeling techniques on different scales, which can facilitate the prediction of transformation plasticity, kinetics and final texture of stress-affected bainite. On the atomic scale we demonstrate an ab initio method of prediction of the parameters for large elastic deformation. Different phase field models from the length scale of carbides to the length scale of a statistically representative set of austenite grains are introduced and discussed. On the largest scale, a macroscopic autocatalytic transformation model with mean field description is used to simulate the evolution of bainite fraction under external tensile stress. These models, in combination with experimental investigations, shall serve as a basis for a deeper understanding and quantitative prediction of the stress-affected bainite transformation in the future.

## Data Availability

The data presented in this study are available on request from the corresponding author.
